# IL-1 Polymorphism and *Helicobacter pylori* Infection Features: Highlighting VNTR’s Potential in Predicting the Susceptibility to Infection-Associated Disease Development

**DOI:** 10.3390/microorganisms11020353

**Published:** 2023-01-31

**Authors:** Hajar El Filaly, Ahmed Outlioua, Christophe Desterke, Zerif Echarki, Wafaa Badre, Moncef Rabhi, Myriam Riyad, Damien Arnoult, Abdelouahed Khalil, Khadija Akarid

**Affiliations:** 1 Biochemistry, Biotechnology and Immunophysiopathology Research Team, Health and Environment Laboratory, Ain Chock Faculty of Sciences, Hassan II University of Casablanca, Casablanca 20100, Morocco; 2 INSERM UMRS-1311, Faculty of Medicine, University of Paris-Saclay, 94270 Villejuif, France; 3 Research Center on Aging, Faculty of Medicine and Health Sciences, Department of Medicine, University of Sherbrooke, Sherbrooke, QC J1H 5N4, Canada; 4 Gastroenterology Department, CHU Ibn Rochd, Casablanca 20100, Morocco; 5 Diagnostic Center, Hôpital Militaire d’Instruction Mohammed V, Mohammed V University, Rabat 10045, Morocco; 6 Research Team on Immunopathology of Infectious and Systemic Diseases, Laboratory of Cellular and Molecular Pathology, Faculty of Medicine and Pharmacy, UH2C, Casablanca 20250, Morocco; 7 INSERM, UMR_S 1197, Hôpital Paul Brousse, Université Paris-Saclay, 94270 Villejuif, France

**Keywords:** *Helicobacter pylori*, IL-1β, IL-1 polymorphism, rs1143627, rs16944, IL1RN VNTR

## Abstract

Genetic polymorphisms at the IL-1 cluster are associated with increased *Helicobacter pylori* (*H. pylori*)-associated disease risk in an ethnically dependent manner. Due to the corroborated role of IL-1β in *H. pylori* infection progression, our aim is to depict the impact of IL1B rs1143627 and rs16944 as well as the IL1RN variable number of identical tandem repeats (VNTR) on the clinical and biological features of Moroccan *H. pylori*-infected patients. A total of 58 patients with epigastralgic pain were referred to the gastroenterology department for histopathological and clinical analysis. DNA extraction from antrum and fundus biopsies and PCR–RFLP were performed to detect polymorphisms. As a result, VNTR was significantly associated with IL-1β antrum levels (*p*-value = 0.029), where the *1/*4 genotype showed a positive association with upregulated cytokine levels in the antrum and was clustered with *H. pylori*-infected patients’ features and higher levels of IL-1β in the antrum and fundus. Likewise, *1/*1 genotype carriers clustered with severe gastritis activity and *H. pylori* density scores along with low levels of IL-1β in the antrum and fundus, while the *1/*2 genotype was clustered with non-infected-patient features and normal IL-1β levels. In conclusion, VNTR might be an interesting predictor to identify patients at risk of developing *H. pylori*-associated pathologies.

## 1. Introduction

*Helicobacter pylori* (*H. pylori*) is a spiral gram-negative bacterium with flagella, classified by the World Health Organization (WHO) as a class I carcinogen [1,2]. *H. pylori* infects the stomach of half the world’s population and almost two-thirds of people in developing countries. This infection varies by country, age, ethnicity, promiscuous conditions, and hygiene. It can trigger a plethora of pathologies from overlying simple asymptomatic gastritis to more serious pathologies such as gastric cancer [3]. These pathologies are related to the severity of the inflammation and depend on the complex interaction between *H. pylori*, host immune response and environmental factors [4]. In Morocco, this infection presents a prevalence of 69.2% and represents a major public health problem [5].

*H. pylori*-induced inflammatory response results in the secretion of a wide panel of inflammatory cytokines by epithelial and immune cells, such as interleukin-1β (IL-1β), which is a pleiotropic pro-inflammatory cytokine. IL-1β is involved in the initiation and amplification of the inflammatory response during *H. pylori* infection, as well as in the modulation of the secretory function of gastric epithelial cells (CEG), acting as a potent inhibitor of stomach acid secretion, which is necessary for the digestion and elimination of ingested bacteria [6,7]. The overexpression of IL-1β was recently identified as a signature of the intestinal metaplasia stage in the fundus of *H. pylori*-infected patients, an advanced stage that could progress toward gastric cancer [8].

**Figure 1:**
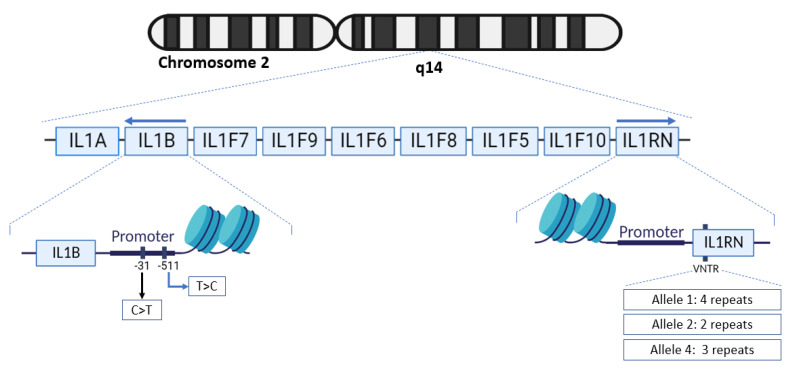
Representation of the IL1 genes cluster, where genes are represented by the boxes and placements of polymorphisms of interest are visualized. In the figure, distances of each gene are not representative of their actual length.

On the chromosome 2q14.2, the IL1 gene cluster is subject to different polymorphisms (Figure 1). This cluster comprises IL1A, IL1B, IL1F7, IL1F9, IL1F6, IL1F8, IL1F5, IL1F10 and IL1RN. Previous studies have demonstrated that the single-nucleotide polymorphism (SNP) at the −31 and −511 positions of the IL-1β gene, IL-1B-31 C/T (rs1143627) and IL-1B-511 T/C (rs16944), as well as the Interleukin-1 receptor antagonist (IL-Ra) gene’s variable number of identical tandem repeats (VNTR) influence IL-1β expression and might predict the prognosis of *H. pylori*-infected patients [9]. Furthermore, epidemiological studies have emphasized that ethnicity can greatly impact the level of this cytokine’s secretion and its involvement in the *H. pylori*-associated physiopathology through various polymorphisms [9,10,11]. Controversially, certain populations have shown no significant association between polymorphisms and *H. pylori*-associated pathologies [12,13].

To our knowledge, no studies have investigated the association of IL-1β polymorphisms and the evolution of *H. pylori* infection in the Moroccan population. Consequently, the main objective of this work is to determine the impact of the three IL-1-associated polymorphisms cited above on Moroccan *H. pylori*-infected patients.

## 2. Material and Methods

### 2.1. Ethics Statement

This research was conducted according to the principles set out in the Declaration of Helsinki and in local ethical guidelines. This study was approved by the Ethics Committee for Biomedical Research, Ibn Rochd Hospital, Casablanca, Morocco. Written informed consent was obtained from all patients.

### 2.2. Patients

Fifty-eight patients referred for an upper gastrointestinal endoscopy to diagnose the cause of abdominal pain were recruited by the Gastroenterology Department of Ibn Rochd Hospital at Casablanca and the military hospital in Rabat, Morocco.

Patients who had undergone previous gastroduodenal surgery, who had been treated to eradicate *H. pylori* or who had taken proton-pump inhibitors or anti-inflammatory drugs during the 3 months preceding the endoscopy were excluded from the present study. The medical history was obtained from each patient included in our study. A total of 6 biopsies were taken from each patient, 3 from the antrum and 3 from the fundus. The biopsies were separately processed to perform histopathological analyses and extract DNA. The tissue sampling procedures as well as the method of sample handling were identical for the biopsies from the antrum and the fundus.

### 2.3. Histopathological Analyses

Routine histopathological analyses were carried out on the gastric mucosal tissues in the anatomopathological department of the CHU Ibn Rochd at Casablanca or the military hospital in Rabat and were assessed according to the updated Sydney system [14]. Briefly, the gastric mucosal tissue samples were fixed in 10% formalin for 24 h and embedded in paraffin. Sequential 3–5 μm sections were cut and stained with hematoxylin–eosin and modified Giemsa.

### 2.4. DNA Extraction

Biopsies intended for the genomic DNA extraction were homogenized using a spatula in sterile Eppendorf tubes and then lysed with lysis buffer containing 20µL of proteinase K at 20 mg/mL (600 U/mL) overnight (14 h) at 56 °C. Genomic DNA isolation from cell lysates was carried out using the standard phenol–chloroform protocol.

### 2.5. PCR–RFLP Assay

The study of the IL1 cluster gene polymorphisms, IL1B at positions −31 and −511, was carried out through restriction enzyme digestion, while IL1RN VNTR was detected with PCR. The primers used for the three polymorphisms are cited in Table 1. The PCR was carried out in a final volume of 25 μL containing 150 ng of DNA, 0.4 µM of each primer (New England Biolabs), 10 mM dNTP (New England Biolabs) and 1 U Taq-polymerase (One Taq, New England biolab). The PCR program for the amplification of the two SNPs was, in turn, started with denaturation at 94 °C for 5 min, 94 °C for 30 s, 56 °C for 30 s, 72 °C for 30 s (35 cycles) and, finally, at 72 °C for 5 min. The digestion was performed by both restriction enzymes avaI (C|YCGRG) for the SNP at position −511 and aluI (AG|CT) for the SNP at position −31. A total of 15 μL of the amplified products was digested with 5U of one of the restriction enzymes (New England Biolabs) at 37 °C for 3 h. Visualization was carried out on 3% agarose gel. The genotypes were analyzed according to the visualized fragments (Table 2).

The analysis of VNTR at the IL1RN level was performed with PCR starting with denaturation at 95 °C for 5 min followed by 35 cycles at 95 °C for 30 s, at 50 °C for 30 s and at 72 °C for 30 s and, lastly, elongation at 72 °C for 5 min. The different alleles were identified according to the size of the fragment amplified. The 410 bp band was classified as allele 1, the 240 bp band was classified as allele 2, the 500 bp band was classified as allele 3, the 325 bp band was classified as allele 4 and the 595 bp band was classified as allele 5. The amplicons were visualized on a 2% agarose electrophoresis gel in the presence of 2 μL BET under ultraviolet light.

### 2.6. Statistical Analysis

Statistical analyses were performed in R software environment version 4.2.1. To determine the patient distribution, a detailed table (Table 3) including all the variables of the study was generated using the “crosstable” function, and the differences’ significance was analyzed through a *t*-test and χ2 test for age and other categorical variables, respectively.

“Chi2loop” R-package version 1.1.2 was used to perform iteration chi-square testing between the categorical variables from the cohort dataset, available on https://github.com/cdesterke/chi2loop (accessed on 21 September 2022). Data must be converted to a data frame before running the following functions. Then, the “cltest” function was applied to perform chi-square iterations between the overall categorical variables, each represented by a character column. The output was filtered and visualized with an NLP plot (NLP: negative log10 of chi-square test *p*-values) through the “nlpplot” function, where only significant associations were represented. The output was also visualized with variable community detection with a Louvain classification algorithm network through the “chinet” function to detect the variables’ clusters.

To determine the eventual association type between polymorphisms and the clinical and biological features of *H. pylori* infection, a Mosaic plot [16] was performed through the “mosaic” function available in the “vcd” package.

At last, and based on the iterative chi-square test, the linked variables to the polymorphisms’ cluster were selected, and a multiple correspondence analysis (MCA) [17] was built with FactoMineR R-package version 2.4. The output was visualized using variable and grouped map representations to differentiate between the *H. pylori*-infected patients’ profiles in regards to their exhibited polymorphisms.

## 3. Results

The patient distribution and polymorphism association with the clinical and biological features of *H. pylori* infection were identified. IL-1B-31 and IL-1B-511 SNPs were respectively identified as rs_31 and rs_511 for the analysis. IL-1β expression data were retrieved from our previous work [8] and were converted into five quantiles (IL-1BQ). The patient distribution according to *H. pylori* infection is presented in Table 3. No significant difference between the numbers of infected and uninfected patients is shown in regard to gender distribution (*p*-value = 0.52), the region of the patient’s residency (urban vs. rural) (*p*-value = 0.57) or smoking habits (*p*-value = 0.26). As for clinical parameters, the *H. pylori* density score and atrophy and metaplasia scores show significant differences between the infected and uninfected patients (respective *p*-values = 1.57 × 10^−12^, 0.047, 0.052). Gastritis activity shows no difference between the infected and non-infected patients (*p*-value = 0.14). Regarding direct relations between infection and the three genetic polymorphisms IL1B-31 SNP, IL1B-511 SNP and VNTR, no significance was observed (respective *p*-values = 0.73, 0.26, 0.59).

**Figure 2:**
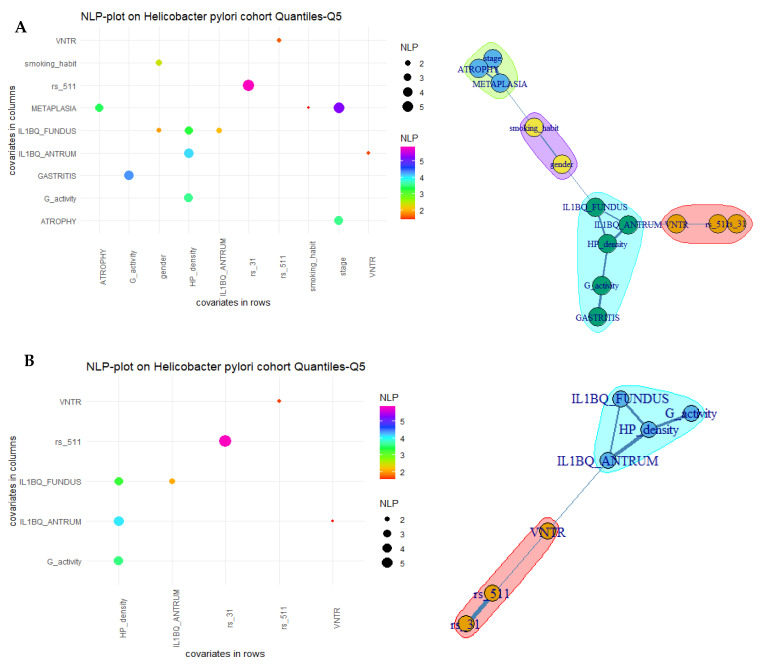
Iterative chi-square tests between categorical variables of the study: (**A**) negative log10 *p*-value plot (NLP plot) and Louvain clustering network including clinical, epidemiological, genetic and IL-b parameters; (**B**) negative log10 *p*-value plot (NLP plot).

In order to determine whether both SNPs and VNTR are associated with other parameters such as the characteristics of the patients and the clinical and biological features of *H. pylori* infection, univariate iterative chi-square tests were performed with all these categorical variables cited in Table 3. According to the NLP plot, IL1B-511 SNP was significantly associated with the other polymorphisms: a great association was noted with IL1B-31 SNP (*p*-value = 1.30 × 10^6^, Figure 2A, Supplementary Table S1) and to a lesser extent with VNTR (*p*-value = 0.024, Figure 2A, Supplementary Table S1). VNTR was found to be significantly associated with IL-1β levels in the antrum (*p*-value = 0.029, Figure 2A, Supplementary Table S1). In the network, the overall polymorphisms were clustered together (pink) and were linked to the cluster with both the clinical and biological features of the infection (light blue cluster) through VNTR (Figure 2A). No direct relations were found between genetic polymorphisms and epidemiologic parameters like smoking habit and gender (Figure 2A). The iterative test was processed again, only using variables within the cluster (light blue cluster) associated with the three polymorphisms to detect the variable communities with a Louvain classification algorithm and confirm the antrum IL-1β levels’ direct association with VNTR (Figure 2B, Supplementary Table S2).

**Figure 3:**
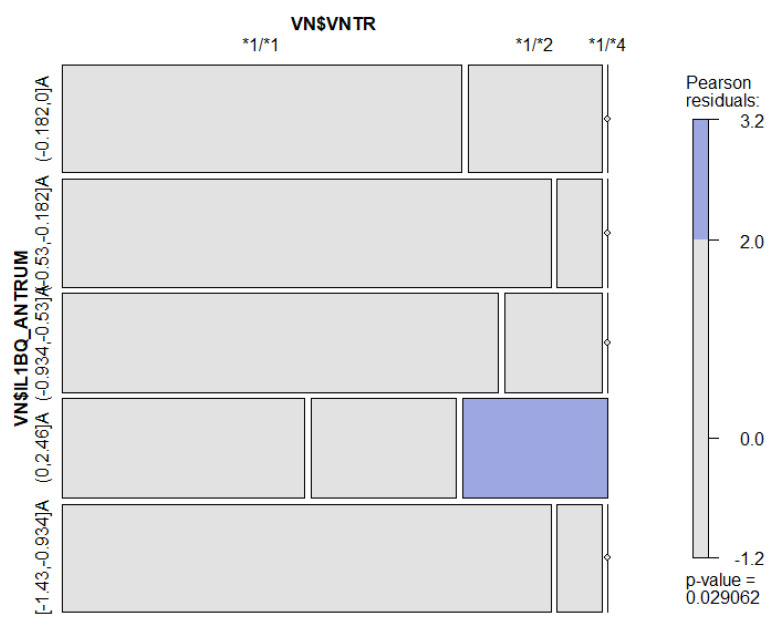
Mosaic plot performed on VNTR genotypes and antrum IL-1β levels. Blue-colored tiles reflect a positive association while grey tiles reflect null association.

Because of the association between VNTR and IL-1β at the antrum level, a Mosaic plot was performed to depict the nature of this association (Figure 3). As a matter of fact, higher levels of antrum IL-1β were significantly linked to the *1/*4 genotype (*p*-value = 0.029).

### Genotypes Carrier Stratification According to Clinical and Biological Features

**Figure 4:**
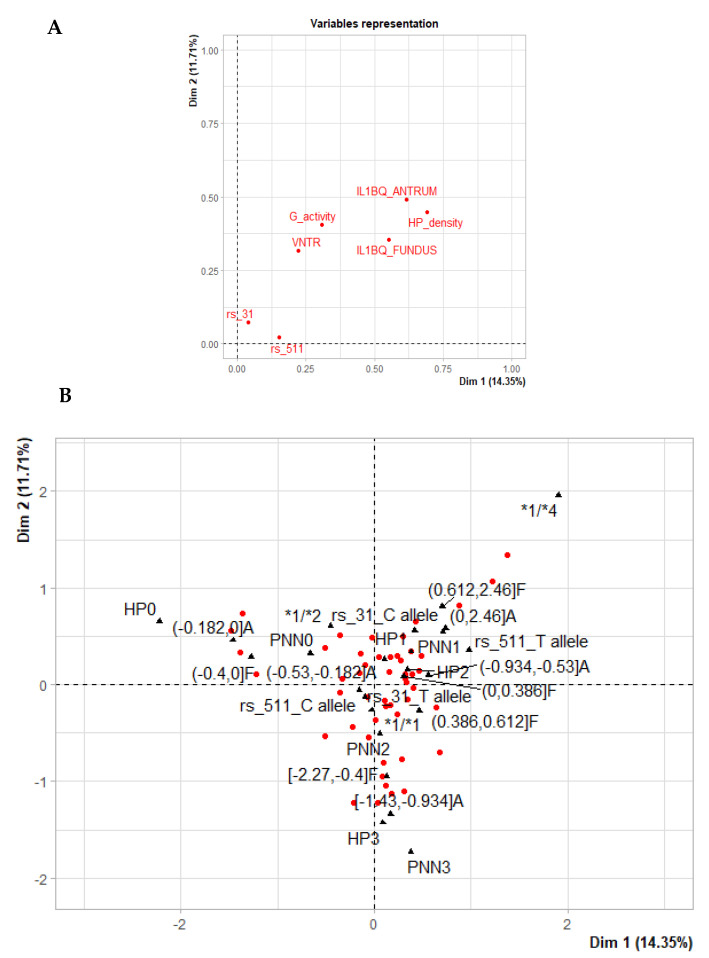
Multiple correspondence analysis between polymorphism and associated parameters: (**A**) variable representation during the MCA; (**B**) distribution of the groups during MCA analysis on the first factorial map.

For further insight, an MCA was performed including the variables associated with the polymorphism cluster, which were the IL-1β levels in both the antrum and fundus and *H. pylori* density and gastritis activity scores (Figure 2B), and was built accordingly (Figure 4A,B). Interestingly, the IL1B-511 T and IL1B-31 C alleles were clustered with low to moderate scores, with higher IL-1β fundic levels. As for VNTR, *1/*2 clustered with non-infected patient features and both negative gastritis activity and *H. pylori* density scores (PNN0 and HP0, respectively). The *1/*4 genotype was clustered with patients presenting low gastritis activity scores (PNN1) and both low and moderate *H. pylori* density scores (HP1 and HP2, respectively) with higher levels of IL-1β in the antrum and the fundus. The *1/*1 genotype was closely situated to the cluster of patients with moderate and severe gastritis activity scores (PNN2 and PNN3, respectively), severe *H. pylori* density scores (HP3) and low levels of IL-1β expression in the antrum and fundus (Figure 4).

These results suggest that both IL1B-511 T and IL1B-31 C allele carriers exhibit a range of low to moderate clinical scores with higher fundic levels of IL-1β. Most importantly, VNTR genotypes might stratify patients through their clinical/biological parameters such as IL-1β levels, gastritis activity and *H. pylori* density scores.

## 4. Discussion

IL-1β is a pro-inflammatory cytokine whose overexpression is closely linked to the development of gastric cancer in *H. pylori*-infected patients [18]. This association is attributed to its ability to inhibit gastric acid production and to bolster inflammation, thereby progressing from gastritis towards atrophy, intestinal metaplasia and gastric cancer [6]. According to several studies, some patients are at higher risk to develop this cancer. This predisposition is explained by an allelic variability induced by the polymorphisms influencing IL-1β expression, which in turn influences the severity of *H. pylori* infection according to the studied population [9]. In fact, IL-1β polymorphism seems to impact the clinical/biological features of *H. pylori* infection in an ethnic manner [19,20]. Therefore, we aim to determine the impact of IL-1-associated polymorphisms (IL-1B SNPs at −511 and −31 positions as well as IL1RN VNTR) within a sample of Moroccan patients exhibiting *H. pylori* infection.

Our results show that, through iterative chi-square testing, no association was observed between both SNPs IL1B-31/IL1B-511 and the clinical/biological features of *H. pylori* infection. The homozygotic T −511 SNP allele has been shown to be associated with higher gastritis activity scores, while no impact has been shown on *H. pylori* density or pathogenesis scores [20]. For better insight, an MCA was built, and its visualized results show that IL1B-511 T and IL1B-31 C allele carriers exhibit a range of low to moderate clinical scores with higher fundic levels of IL-1β. In our previous work, we showed that higher levels of fundic IL-1β are a signature of metaplasia in *H. pylori*-infected Moroccan patients [8]. This advanced stage has been depicted through advanced research as a point of no return regarding gastric cancer, highlighting the need for early intervention against *H. pylori* infection to diminish the risk of developing this neoplasia [21]. These results suggest that these two allele carriers might be susceptible to developing *H. pylori*-associated pathologies, which might progress towards advanced stages because of the higher IL-1β levels exhibited by these patients. Other studies have shown that IL1B-511 T allele carriers exhibit an overexpression of IL-1β in the antrum and fundus of Japanese and German patients following infection by *H. pylori* [20,22]. As a matter of fact, the IL1B-511 T allele has been linked to severe gastric inflammation and gastric cancer within Brazilian and Italian populations [23,24,25]. Similarly, Taiwanese carriers of the IL-1B-511 T allele have an increased risk of reflux esophagitis [26]. Nevertheless, Murphy et al. found no evidence linking a higher risk of developing *H. pylori*-associated diseases with IL-1B-511 SNP in the Irish population [27]. As for IL-1B-31 SNP, studies on the Brazilian population suggest that IL1B-31 C allele carriers may have enhanced IL-1β production at the gastric level, heightening the risk of developing severe chronic gastritis and gastric carcinoma [24,25]. This allele has also been associated with a higher risk of reflux esophagitis within the Taiwanese population [26].

As for VNTR, patients with different genotypes exhibited distinctive clinical/biological parameters (IL-1β levels, gastritis activity, *H. pylori* density score). In addition, this polymorphism was linked to IL-1β at the antrum level, where the *1/*4 genotype showed a positive association with higher levels of this cytokine in the antrum. At this level, patients with *H. pylori*-induced gastritis exhibiting no advanced stage showed higher levels of IL-1β when compared to non-infected patients [28,29]. In our data, higher levels of IL-1β at the level of the antrum were exhibited in the early stages such as gastritis (Supplementary Table S3). According to the *H. pylori* infection etiology, the gastric antrum serves as a critical niche for *H. pylori* infection due to its higher pH [7,14]. Other than that, clinical case studies have shown that advanced stages are mostly observed at this level and might eventually progress toward the corpus, which might be explained by this IL-1β upregulation [30]. As a matter of fact, higher IL-1β has been shown to modulate gastrin levels by acting on G cells, resulting in antral transformation [31]. These overall observations might suggest that VNTR is able to determine the susceptibility of developing various *H. pylori*-associated diseases. Remarkably, *1/*4 genotype carriers might be at higher risk of developing antral inflammation through elevated IL-1β expression. The results of previous studies have shown that IL-1Ra polymorphism significantly impacts IL-1β expression [20,22]. Differentially from what we found, *2 allele carriers exhibited higher IL-1β expression with severe degrees of inflammation and a close association with atrophy in the antrum and corpus in Japanese and German populations [20,22]. In the Brazilian population, the *2 allele exhibited higher levels of IL-1β [28]. In the same population, there is an association of this allele variant with susceptibility to chronic gastritis and gastric cancer [32].

These overall results suggest that within this sample of Moroccan patients, these analyzed IL-1 polymorphisms might be a potential predictor of *H. pylori*-associated disease development.

## 5. Conclusions

Because of the response diversity exhibited by *H. pylori*-infected patients, the necessity of finding out predictors will extensively help to prevent advanced-stage development. To our knowledge, the present study is the first to depict the relationship between IL-1 polymorphism and *H. pylori* clinical features in the Moroccan population, which exhibits a higher prevalence of *H. pylori* infection. In fact, ethnicity seems to impact the host response to *H. pylori* infection through polymorphism and might predispose certain patients to develop advanced stages by modulating IL-1β levels. Although a low number of patients were recruited for this study, our results show that IL1B-511 T and IL1B-31 C allele carriers might be susceptible to developing *H. pylori*-associated pathologies. In addition, and to a further interesting extent, VNTR might be a predicting factor by differentiating *H. pylori*-infected patients, pointing out those at risk of developing serious diseases.

## Figures and Tables

**Table 1:** Primers used for PCR.

Polymorphism	Primer Sequences
Rs16944 (−511)	5′-TGGCATTGATCTGGTTCATC-3′
	5′-GTTTAGGAATCTTCCCACTT-3′
Rs1143627 (−31)	5′-AGAAGCTTCCACCAATACTC-3′
	5′-AGCACCTAGTTGTAAGGAAG-3′
IL-1RN VNTR [15]	5′-CCTCAGCAACACTCCTAT-3′
	5′-TCCTGGTCTGCAGGTAA-3′

**Table 2:** Description of visualized fragments after RFLP.

	TT	CT	CC
IL-1B −31 C/T	137 pb + 102 pb	238 pb + 137 pb + 102 pb	238 pb
IL-1B −511 T/C	304 pb	304 pb + 190 pb + 114 pb	190 pb + 114 pb

**Table 3:** Distribution of patients according to *H. pylori* infection status. The scores were accorded as reported in the Sydney classification [14].

		Infection Status N (%)		
		Infected (N = 51)	Non-Infected (N = 7)	*p*-Values
Age mean		45.9	40.6	0.4703
Gender	F	21 (91.30)	2 (8.70)	0.5226
	M	30 (85.71)	5 (14.29)	
Region	Urban	47 (88.68)	6 (11.32)	0.569
	Rural	4 (80.00)	1 (20.00)	
Smoking habit	FS	6 (100.00)	0 (0)	0.2646
	NS	40 (88.89)	5 (11.11)	
	S	5 (71.43)	2 (28.57)	
*H. pylori* density score	HP0	0 (0)	7 (100.00)	1.572 × 10^−12^
	HP1	18 (100.00)	0 (0)	
	HP2	23 (100.00)	0 (0)	
	HP3	10 (100.00)	0 (0)	
Gastritis activity	PNN0	16 (76.19)	5 (23.81)	0.1402
	PNN1	16 (100.00)	0 (0)	
	PNN2	15 (88.24)	2 (11.76)	
	PNN3	4 (100.00)	0 (0)	
−31 SNP (rs_31)	C allele	10 (90.91)	1 (9.09%)	0.7363
	T allele	41 (87.23)	6 (12.77)	
−511 SNP (Rs_511)	C allele	43 (86.00)	7 (14.00)	0.2591
	T allele	8 (100.00)	0 (0)	
VNTR	*1/*1	40 (88.89)	5 (11.11)	0.5935
	*1/*2	8 (80.00)	2 (20.00)	
	*1/*4	3 (100.00)	0 (0)	
Gastritis	Low	12 (85.71)	2 (14.29)	0.6279
	Moderate	33 (86.84)	5 (13.16)	
	severe	6 (100.00)	0 (0)	
Atrophy	None	34 (89.47)	4 (10.53)	0.04721
	Low	13 (86.67)	2 (13.33)	
	Moderate	4 (100.00)	0 (0)	
	severe	0 (0)	1 (100.00)	
Metaplasia	none	46 (92.00)	4 (8.00)	0.05687
	Low	3 (60.00)	2 (40.00)	
	Moderate	2 (66.67)	1 (33.33)	
Stage	Gastritis	34 (82.93)	7 (17.07)	0.2802
	Atrophy	13 (100.00)	0 (0)	
	Metaplasia	4 (100.00)	0 (0)	

## Data Availability

The data presented in this study are available on request.

## Supplementary Materials

The following supporting information can be downloaded at: https://www.mdpi.com/article/10.3390/microorganisms11020353/s1, Table S1: Iterative chi.square testing association between each clinical/biological parameters. Table S2: Iterative chi.square testing association between each clinical/biological parameters. Table S3: Distribution of gastritis according to IL-1β ANTRUM Levels.

## References

[1] Robinwarren J. (1983). Unidentified Curved Bacilli on Gastric Epithelium in Active Chronic Gastritis. Lancet.

[2] Wang F., Meng W., Wang B., Qiao L. (2014). Helicobacter Pylori-Induced Gastric Inflammation and Gastric Cancer. Cancer Lett..

[3] den Hoed C.M., Kuipers E.J., Ryan E.T., Hill D.R., Solomon T., Aronson N.E., Endy T.P. (2020). 45—Helicobacter Pylori Infection. Hunter’s Tropical Medicine and Emerging Infectious Diseases.

[4] Cadamuro A.C.T., Rossi A.F.T., Maniezzo N.M., Silva A.E. (2014). Helicobacter Pylori Infection: Host Immune Response, Implications on Gene Expression and MicroRNAs. World J. Gastroenterol..

[5] Bounder G., Boura H., Nadifiyine S., Jouimyi M.R., Bensassi M., Kadi M., Eljihad M., Badre W., Benomar H., Kettani A. (2017). Epidemiology of Helicobacter Pylori Infection and Related Gastric Pathologies in Moroccan Population. J. Life Sci..

[6] El Filaly H., Outlioua A., Medyouf H., Guessous F., Akarid K. (2022). Targeting IL-1β in Patients with Advanced Helicobacter Pylori Infection: A Potential Therapy for Gastric Cancer. Future Microbiol..

[7] Sáenz J.B., Mills J.C. (2018). Acid and the Basis for Cellular Plasticity and Reprogramming in Gastric Repair and Cancer. Nat. Rev. Gastroenterol. Hepatol..

[8] Outlioua A., Badre W., Desterke C., Echarki Z., El Hammani N., Rabhi M., Riyad M., Karkouri M., Arnoult D., Khalil A. (2020). Gastric IL-1β, IL-8, and IL-17A Expression in Moroccan Patients Infected with Helicobacter Pylori May Be a Predictive Signature of Severe Pathological Stages. Cytokine.

[9] de Brito B.B., da Silva F.A.F., de Melo F.F. (2018). Role of Polymorphisms in Genes That Encode Cytokines and Helicobacter Pylori Virulence Factors in Gastric Carcinogenesis. World J. Clin. Oncol..

[10] Martínez-Carrillo D.N., Garza-González E., Betancourt-Linares R., Mónico-Manzano T., Antúnez-Rivera C., Román-Román A., Flores-Alfaro E., Illades-Aguiar B., Fernández-Tilapa G. (2010). Association of IL1B -511C/-31T Haplotype and Helicobacter Pylori VacA Genotypes with Gastric Ulcer and Chronic Gastritis. BMC Gastroenterol..

[11] Manchanda P.K., Bid H.K., Mittal R.D. (2005). Ethnicity Greatly Influences the Interleukin-1 Gene Cluster(IL- 1β Promoter, Exon-5 and IL-1Ra) Polymorphisms: A Pilot Study of a North Indian Population. Asian Pac. J. Cancer Prev..

[12] Sierra R., Une C., Ramirez V., Alpizar-Alpizar W., Gonzalez M.-I., Ramirez J.-A., De Mascarel A., Cuenca P., Perez-Perez G., Megraud F. (2008). Relation of Atrophic Gastritis with Helicobacter Pylori-CagA(+) and Interleukin-1 Gene Polymorphisms. World J. Gastroenterol..

[13] Moorchung N., Srivastava A.N., Gupta N.K., Ghoshal U.C., Achyut B.R., Mittal B. (2007). Cytokine Gene Polymorphisms and the Pathology of Chronic Gastritis. Singap. Med. J..

[14] Hassan T.M.M., Al-Najjar S.I., Al-Zahrani I.H., Alanazi F.I.B., Alotibi M.G. (2016). Helicobacter Pylori Chronic Gastritis Updated Sydney Grading in Relation to Endoscopic Findings and H. Pylori IgG Antibody: Diagnostic Methods. J. Microsc. Ultrastruct..

[15] Ryberg A., Borch K., Sun Y.-Q., Monstein H.-J. (2008). Concurrent Genotyping of Helicobacter Pylori Virulence Genes and Human Cytokine SNP Sites Using Whole Genome Amplified DNA Derived from Minute Amounts of Gastric Biopsy Specimen DNA. BMC Microbiol..

[16] Friendly M. (2002). A Brief History of the Mosaic Display. J. Comput. Graph. Stat..

[17] Ayele D., Zewotir T., Mwambi H. (2014). Multiple Correspondence Analysis as a Tool for Analysis of Large Health Surveys in African Settings. Afr. Health Sci..

[18] Rébé C., Ghiringhelli F. (2020). Interleukin-1β and Cancer. Cancers.

[19] Hong J.-B., Zuo W., Wang A.-J., Lu N.-H. (2016). Helicobacter Pylori Infection Synergistic with IL-1β Gene Polymorphisms Potentially Contributes to the Carcinogenesis of Gastric Cancer. Int. J. Med. Sci..

[20] Hwang I.-R., Kodama T., Kikuchi S., Sakai K., Peterson L.E., Graham D.Y., Yamaoka Y. (2002). Effect of Interleukin 1 Polymorphisms on Gastric Mucosal Interleukin 1β Production in Helicobacter Pylori Infection. Gastroenterology.

[21] Michigami Y., Watari J., Ito C., Nakai K., Yamasaki T., Kondo T., Kono T., Tozawa K., Tomita T., Oshima T. (2018). Long-Term Effects of H. Pylori Eradication on Epigenetic Alterations Related to Gastric Carcinogenesis. Sci. Rep..

[22] Rad R., Dossumbekova A., Neu B., Lang R., Bauer S., Saur D., Gerhard M., Prinz C. (2004). Cytokine Gene Polymorphisms Influence Mucosal Cytokine Expression, Gastric Inflammation, and Host Specific Colonisation during Helicobacter Pylori Infection. Gut.

[23] Ruzzo A., Graziano F., Pizzagalli F., Santini D., Battistelli V., Panunzi S., Canestrari E., Catalano V., Humar B., Ficarelli R. (2005). Interleukin 1B Gene (IL-1B) and Interleukin 1 Receptor Antagonist Gene (IL-1RN) Polymorphisms in Helicobacter Pylori-Negative Gastric Cancer of Intestinal and Diffuse Histotype. Ann. Oncol..

[24] Ramis I.B., Vianna J.S., Halicki P.C.B., Lara C., Tadiotto T.F., da Maciel J.B.S., Gonçalves C.V., von Groll A., Dellagostin O.A., da Silva P.E.A. (2015). Relationship of Interleukin-1B Gene Promoter Region Polymorphism with Helicobacter Pylori Infection and Gastritis. J. Infect. Dev. Ctries..

[25] Rech T.F., Mazzoleni L.E., Mazzoleni F., de Francesconi C.F.M., Sander G.B., Michita R.T., Nabinger D.D., de Bona L.R., Milbradt T.C., Ott E.A. (2020). Analysis of the Influence of Interleukin-1β Gene Polymorphism on Gastric Inflammatory Response and Precancerous Lesions Development in Patients with Functional Dyspepsia. Immunol. Investig..

[26] Cheng H.-H., Chang C.-S., Wang H.-J., Wang W.-C. (2010). Interleukin-1β and -10 Polymorphisms Influence Erosive Reflux Esophagitis and Gastritis in Taiwanese Patients: Interleukin-1β and -10 in Esophagitis. J. Gastroenterol. Hepatol..

[27] Murphy G., Thornton J., McManus R., Swan N., Ryan B., Hughes D.J., O’Morain C.A., O’Sullivan M. (2009). Association of Gastric Disease with Polymorphisms in the Inflammatory-Related Genes IL-1B, IL-1RN, IL-10, TNF and TLR4. Eur. J. Gastroenterol. Hepatol..

[28] Queiroz D.M.M., Rocha A.M.C., Melo F.F., Rocha G.A., Teixeira K.N., Carvalho S.D., Bittencourt P.F.S., Castro L.P.F., Crabtree J.E. (2013). Increased Gastric IL-1β Concentration and Iron Deficiency Parameters in H. Pylori Infected Children. PLoS ONE.

[29] Serrano C.A., Villagrán A., Toledo H., Crabtree J.E., Harris P.R. (2016). Iron Deficiency and IL1β Polymorphisms in *Helicobacter Pylori* -Infected Children. Helicobacter.

[30] Correa P., Piazuelo M.B., Wilson K.T. (2010). Pathology of Gastric Intestinal Metaplasia: Clinical Implications. Am. J. Gastroenterol..

[31] Ding L., Sontz E.A., Saqui-Salces M., Merchant J.L. (2021). Interleukin-1β Suppresses Gastrin via Primary Cilia and Induces Antral Hyperplasia. Cell. Mol. Gastroenterol. Hepatol..

[32] Oliveira J.G., Duarte M.C., Silva A.E. (2012). IL-1ra Anti-Inflammatory Cytokine Polymorphism Is Associated with Risk of Gastric Cancer and Chronic Gastritis in a Brazilian Population, but the TNF-β pro-Inflammatory Cytokine Is Not. Mol. Biol. Rep..

