# Supplementary material for: IL-1 Polymorphism and Helicobacter pylori Infection Features: Highlighting VNTR’s Potential in Predicting the Susceptibility to Infection-Associated Disease Development
Source: Microorganisms. 2023 Jan 31;11(2):353. doi: 10.3390/microorganisms11020353 (PMC9961292; doi:10.3390/microorganisms11020353)
Supplement: Supplementary file 1 [file microorganisms-11-00353-s001.zip › microorganisms-2122367-supplementary.pdf]

Supplementary material

Supplementary Table S1: Iterative chi.square testing association between each clinical/biological parameters.

First variable	Second Variable	Chi.Square	p.value	NLP
rs_31	rs_511	23.425	1.30E-06	5.88638035
stage	METAPLASIA	29.764	5.47E-06	5.26224544
G_activity	GASTRITIS	29.67	4.54E-05	4.34281237
HP_density	IL1BQ_ANTRUM	39.709	8.04E-05	4.09491711
HP_density	G_activity	31.289	0.00026409	3.57824938
stage	ATROPHY	25.506	0.00027506	3.56057558
ATROPHY	METAPLASIA	24.674	0.00039224	3.40644779
HP_density	IL1BQ_FUNDUS	34.672	0.00052813	3.27726184
gender	smoking_habit	11.011	0.00406478	2.39096346
IL1BQ_ANTRUM	IL1BQ_FUNDUS	32.407	0.00884821	2.05314464
gender	IL1BQ_FUNDUS	12.732	0.01266344	1.89744821
rs_511	VNTR	7.447	0.02415466	1.61699899
VNTR	IL1BQ_ANTRUM	17.102	0.02906241	1.5366683
smoking_habit	METAPLASIA	10.233	0.0366874	1.43548304

Supplementary Table S2: Iterative chi.square testing association between each clinical/biological parameters.

First variable	Second Variable	Chi.Square	p.value	NLP
rs_31	rs_511	23.425	1.30E-06	5.88638035
HP_density	IL1BQ_ANTRUM	39.709	8.04E-05	4.09491711
HP_density	G_activity	31.289	0.00026409	3.57824938
HP_density	IL1BQ_FUNDUS	34.672	0.00052813	3.27726184
IL1BQ_ANTRUM	IL1BQ_FUNDUS	32.407	0.00884821	2.05314464
rs_511	VNTR	7.447	0.02415466	1.61699899
VNTR	IL1BQ_ANTRUM	17.102	0.02906241	1.5366683

Supplementary Table S3: Distribution of gastritis according to IL-1β ANTRUM Levels.

	IL-1β ANTRUM Levels N (%)				
	[-1.43, -0.934]A	(-0.934, -0.53]A	(-0.53, -0.182]A	(-0.182, 0]A	(0, 2.46]A
Gastritis	5 (12.20)	7 (17.07)	9 (21.95)	10 (24.39)	10 (24.39)
